# The role of reactive oxygen species and subsequent DNA-damage response in the emergence of resistance towards resveratrol in colon cancer models

**DOI:** 10.1038/cddis.2014.486

**Published:** 2014-11-20

**Authors:** D J Colin, E Limagne, K Ragot, G Lizard, F Ghiringhelli, É Solary, B Chauffert, N Latruffe, D Delmas

**Affiliations:** 1 Université de Bourgogne, Dijon F-21000, France; 2 Centre de Recherche Inserm U866—Lipids, Nutrition, Cancers, Dijon F-21000, France; 3 EA7270—Bio-PeroxIL Biochimie du peroxysome, inflammation et métabolisme lipidique, Dijon F-21000, France; 4 Centre de Lutte Contre le Cancer GF Leclerc, Dijon F-21000, France; 5 Inserm UMR 1009, Institut Gustave Roussy, 114 rue Edouard Vaillant, 94805 Villejuif cedex, France

## Abstract

In spite of the novel strategies to treat colon cancer, mortality rates associated with this disease remain consistently high. Tumour recurrence has been linked to the induction of resistance towards chemotherapy that involves cellular events that enable cancer cells to escape cell death. Treatment of colon cancer mainly implicates direct or indirect DNA-damaging agents and increased repair or tolerances towards subsequent lesions contribute to generate resistant populations. Resveratrol (RSV), a potent chemosensitising polyphenol, might share common properties with chemotherapeutic drugs through its indirect DNA-damaging effects reported *in vitro*. In this study, we investigated how RSV exerts its anticancer effects in models of colon cancer with a particular emphasis on the DNA-damage response (DDR; PIKKs-Chks-p53 signalling cascade) and its cellular consequences. We showed *in vitro* and *in vivo* that colon cancer models could progressively escape the repeated pharmacological treatments with RSV. We observed for the first time that this response was correlated with transient activation of the DDR, of apoptosis and senescence. *In vitro*, a single treatment with RSV induced a DDR correlated with S-phase delay and apoptosis, but prolonged treatments led to transient micronucleations and senescence phenotypes associated with polyploidisation. Ultimately, stable resistant populations towards RSV displaying higher degrees of ploidy and macronucleation as compared to parental cells emerged. We linked these transient effects and resistance emergence to the abilities of these cells to progressively escape RSV-induced DNA damage. Finally, we demonstrated that this DNA damage was triggered by an overproduction of reactive oxygen species (ROS) against which cancer cells could adapt under prolonged exposure to RSV. This study provides a pre-clinical analysis of the long-term effects of RSV and highlights ROS as main agents in RSV's indirect DNA-damaging properties and consequences in terms of anticancer response and potent resistance emergence.

In spite of chemotherapy and systematic screening for people at risk, the mortality rate of colorectal cancer (CCR) remains high and stable, with 6 00 000 deaths per year. The 5-year survival ranges from 90% in patients with stage I to 10% in patients with stage IV disease.^1^ This low success rate in the treatment of CCR results from many failures associated with high resistance and the risk of metastasis. Resistance has been related to the adaptations that inhibit the ability of tumour cells to die by apoptosis. These adaptations are well known to implicate other mechanisms that control the pro-survival/pro-apoptotic balance including autophagy and senescence.^2, 3^ Senescence constitutes a powerful resistance mechanism towards CCR chemotherapy, which typically involves DNA-damaging agents nowadays.^4^ Indeed, cancer cells respond to DNA damage by initiating the DNA-damage response (DDR), which induces cell cycle delay, more prolonged growth arrests (senescence) and apoptosis of the lethally damaged cells. The DDR is initiated by the phosphatidylinositol 3-kinase-related kinases (PIKKs) ATM, ATR and DNA-PKcs that elicit DNA repair, concomitant cell cycle progression and apoptosis regulations through the Chk1/2-p53-p21 signalling cascade.^5^

One promising approach to counteract chemoresistance could be the use of adjuvants. Among them, resveratrol (trans-3,4′,5-trihydroxystilbene; RSV) has been shown to prevent or delay the different steps of carcinogenesis^6, 7^ through its capacity to inhibit cell cycle progression, to induce apoptosis,^8, 9^ autophagic-related cell death^10^ and senescence *via* DNA damage.^11, 12^ Because of its low toxicity in animal models^6^ and in humans^13, 14, 15^, RSV has been proposed as a potent adjuvant to sensitise cancer cells to various anticancer drugs,^16, 17, 18^ cytokines (e.g. TRAIL),^19^ and ionising radiation.^20^ Recently, phase I/II clinical trials have shown that administration of RSV was correlated with a 5% reduction of tumour growth in patients with confirmed CCR, despite its low bioavailability in its unmetabolised form.^21^ Accordingly, we have proved *in vitro* that RSV metabolites were able to induce the DDR, subsequent S-phase delay and apoptosis and improved the efficacy of anticancer drugs.^22^

Considering that RSV behave as an apparent DNA-damaging agent, we investigated its long-term effects towards CCR models with a particular emphasis on its DNA-damaging properties and the related consequences *in vitro* and *in vivo* in terms of resistance. We demonstrated that RSV-induced DNA damage was triggered by an overproduction of reactive oxygen species (ROS) against which cancer cells could adapt under prolonged exposure to RSV. These effects have to be considered pre-clinically to further point out RSV as a potent chemosensitising agent.

## Results

### Transient DNA-damage response is associated with a resistance towards resveratrol treatments in models of CCR *in vivo*

**Figure 1:**
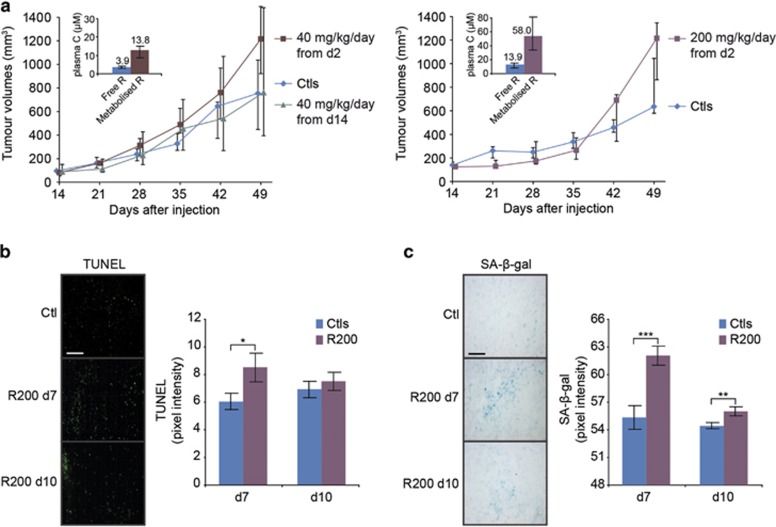
Resveratrol promotes transient accelerated senescence and apoptosis *in vivo*. (**a**) Effects of daily oral administrations of 40 and 200 mg/kg of RSV on the growth of PROb tumours *in vivo*. PROb cells were subcutaneously injected at day 0 to BD-IX rats and the treatments with RSV or with the vehicle (Ctl) began at day 2 or 14. Data are medians of tumour volumes of nine rats per group±the first and third quartile of one representative experiment among three. No statistically significant differences were found by the Mann–Whitney's test. Inserts show the median±the first and third quartile of plasma concentrations of free and metabolised RSV measured 1 h after the last administration of RSV to the rats of the experiment shown. (**b**) Microscopic evaluation of DNA fragmentation by TUNEL staining in PROb tumours. Pixels intensities were measured on 10 random microscopic fields of five tumours per group and is plotted as mean intensity±S.D. and compared with control tumours (Ctl). (**c**) Microscopic evaluation of SA-*β*-galactosidase activity in PROb tumours resected after 7 and 10 days of daily treatments with 200 mg/kg of RSV (R200). Negative pixels intensities measurement related to SA-*β*-galactosidase staining were performed as described above. Typical microscopic fields are shown on the left; *bar*, 100 *μ*m. On both panels, statistical significance was determined by the Student's *t*-test with *P*<0.05 (*), *P*<0.01 (**) and *P*<0.001 (***)

First, we determined the effects of the daily administrations of pharmacological doses of RSV to syngeneic rats bearing subcutaneous PROb CCR. Our results showed that RSV failed to delay the growth of tumours at 40 mg/kg per day either by beginning the treatments 2 or 14 days after the injection of PROb cells (Figure 1a). To further investigate this controversial result, a higher dose of 200 mg/kg per day was used and did not statistically interfere with tumour growth as well. Overall, these first results showed that RSV effects towards PROb tumours did not agree with other studies, although the doses used produced plasma levels of free and metabolised RSV compatible with anticancer effects reported on CCR^22^ and on other models^6^ (Figure 1a, inserts).

To further explore the unexpected response of this *in vivo* model, we assessed if this apparent lack of effect was associated with an absence of early macroscopic effects in tumours. Surprisingly, after 7 and 10 days of treatments, a significant but transient induction of apoptosis was observed (Figure 1b). Also, senescence was robustly induced by RSV (Figure 1c). This increase strongly diminished with the time of treatment while remaining significant compared with the control.

**Figure 2:**
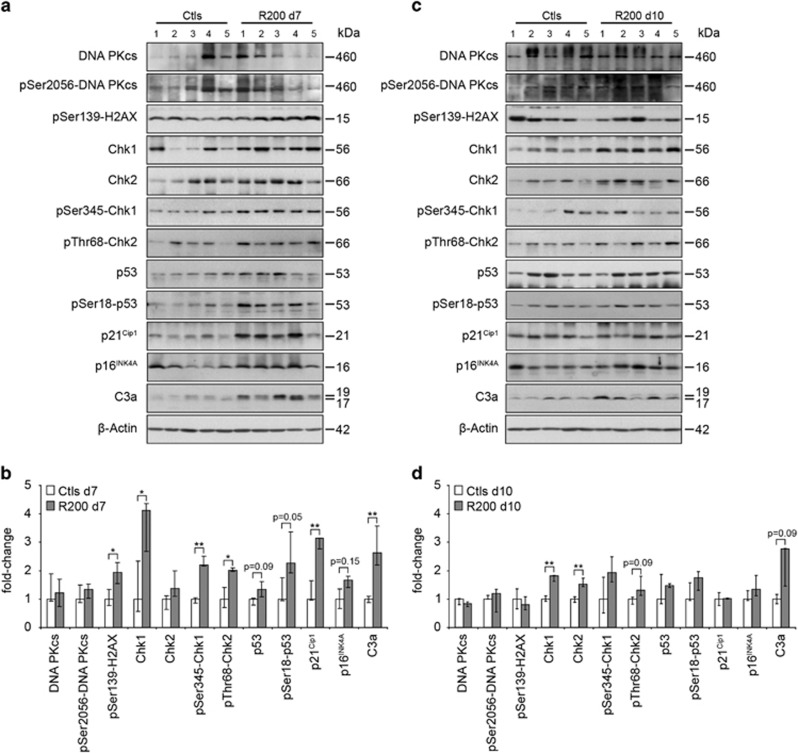
Resveratrol induces a transient activation of the DNA-damage response *in vivo*. Levels of expression and phosphorylation of the components of the DNA-damage response were analysed by immunoblotting using the specified antibodies. Analyses were performed on PROb tumours extracts collected from five rats per group after 7 (**a**) and 10 days (**c**) of daily treatments with 200 mg/kg of RSV (R200) and compared to control tumours (Ctl). Quantifications by densitometry (**b**, **d**) are plotted as medians±the first and third quartile. Statistical analyses were performed by the Mann–Whitney's test and are shown as significant with *P*<0.05 (*) and *P*<0.01 (**)

Transient inductions of apoptosis and senescence have been correlated with the emergence of tumour resistance towards a plethora of therapies, including DNA-damaging agents.^2, 3^ Here, after 7 days of treatment, RSV induced an overall activation of the DDR (Figures 2a and b). We observed an increase of the phosphorylation of the histone H2AX (*γ*H2AX), which is a marker of single and double-stranded DNA breaks. Indeed, the PIKKs (ATM, ATR and DNA-PK) phosphorylate this histone to prime damaged DNA for repair and initiate the DDR. In agreement, RSV further induced the cascade of phosphorylations involving the checkpoint kinases Chk1 and Chk2 and ultimately leading to the stabilisation of p53 through its phosphorylation on its Ser18. Thus, p53 played its transcription factor role as shown by the overexpression of its target p21 in treated tumours. p21 is known to be implicated in the cell death–cell survival balance and its overexpression has also been linked to senescence induction concomitantly with the important mediator p16.^23^ Accordingly, p16 was slightly overexpressed in RSV-treated tumours. RSV-induced caspase-3 activation (Figure 2) confirmed apoptosis induction (Figure 1b). Importantly, these activations were transient, since after 10 days of treatment most of these inductions became statistically undetectable (Figures 2c and d).

**Figure 3:**
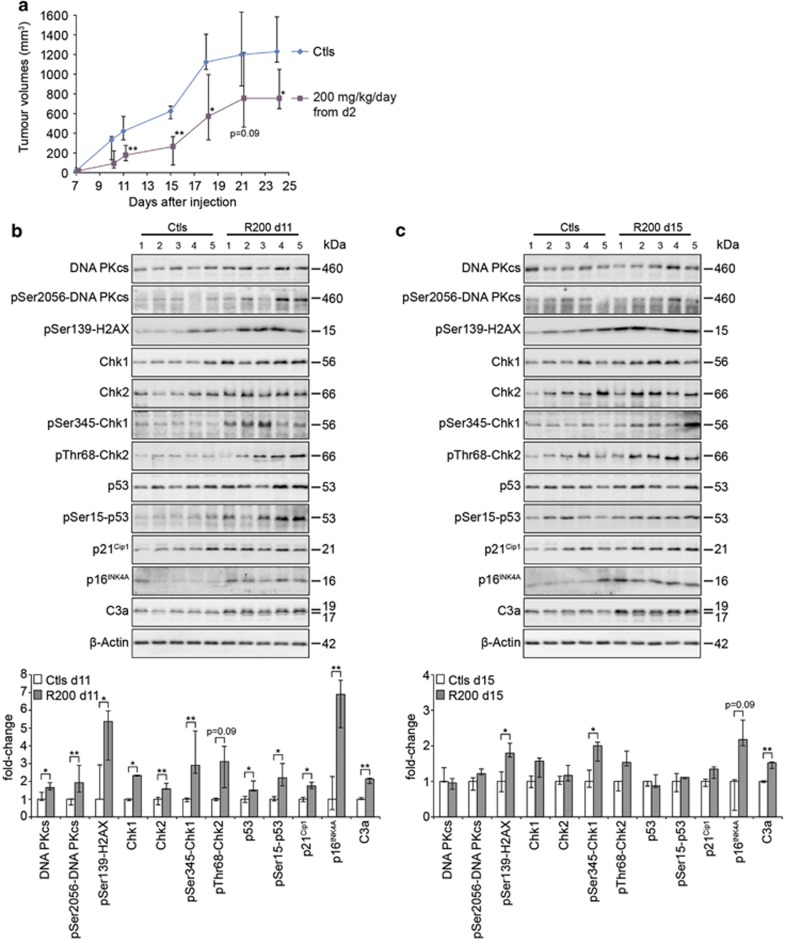
Resveratrol induces a transient activation of the DNA-damage response *in vivo*. (**a**) Effects of daily oral administrations of 200 mg/kg of RSV on the growth of SW620 tumours *in vivo*. SW620 cells were subcutaneously injected at day 0 to nude mice and the treatments with RSV or with the vehicle (Ctl) began at day 2. Data are medians of tumour volumes of 9–12 mice per group±the first and third quartile. Statistical analyses were performed by the Mann–Whitney's test and are shown as significant with *P*<0.05 (*) and *P*<0.01 (**). (**b**, **c**) Levels of expression and phosphorylation of the components of the DNA-damage response were analysed by immunoblotting using the specified antibodies. Analyses were performed on SW620 tumours extracts collected from five mice per group after 11 (**b**) and 15 days (**c**) of daily treatments with 200 mg/kg of RSV (R200) and compared to control tumours (Ctl). Quantifications by densitometry are plotted as medians±the first and third quartile. Statistical analyses were performed by the Mann–Whitney's test and are shown as significant with *P*<0.05 (*) and *P*<0.01 (**)

These results correlating transient DDR, apoptosis and senescence activations by RSV led us to explore the response of a more standard model of human CCR xenograft in Nude mice. We used the SW620 model which has already exhibited sensitivity towards RSV *in vitro* (Supplementary Table S1). In contrast to the PROb model, RSV treatment led to a growth delay of SW620 tumours (Figure 3a), showing the model-dependent macroscopic effects of RSV. Interestingly, the extent of this delay appeared to decrease with time according to the statistical analysis. We biochemically characterised the response of this model and found a similar trend in terms of DDR induction as compared with the PROb model (Figures 3b and c). Indeed, all the markers described earlier were induced in RSV-treated tumours, including the senescence p16 and apoptosis caspase-3 markers. Importantly, after 15 days of treatment, most of these inductions were compromised, which agreed with what we found in the PROb model.

Overall, these results demonstrate that *in vivo* models of CCR treated by RSV can progressively escape its pro-apoptotic and pro-senescence effects, and that this resistance phenomenon is correlated with a short-term DDR induction.

### Repeated treatments with resveratrol induce a polyploidisation and an escape of colon cancer cells from its antiproliferative effects

**Figure 4:**
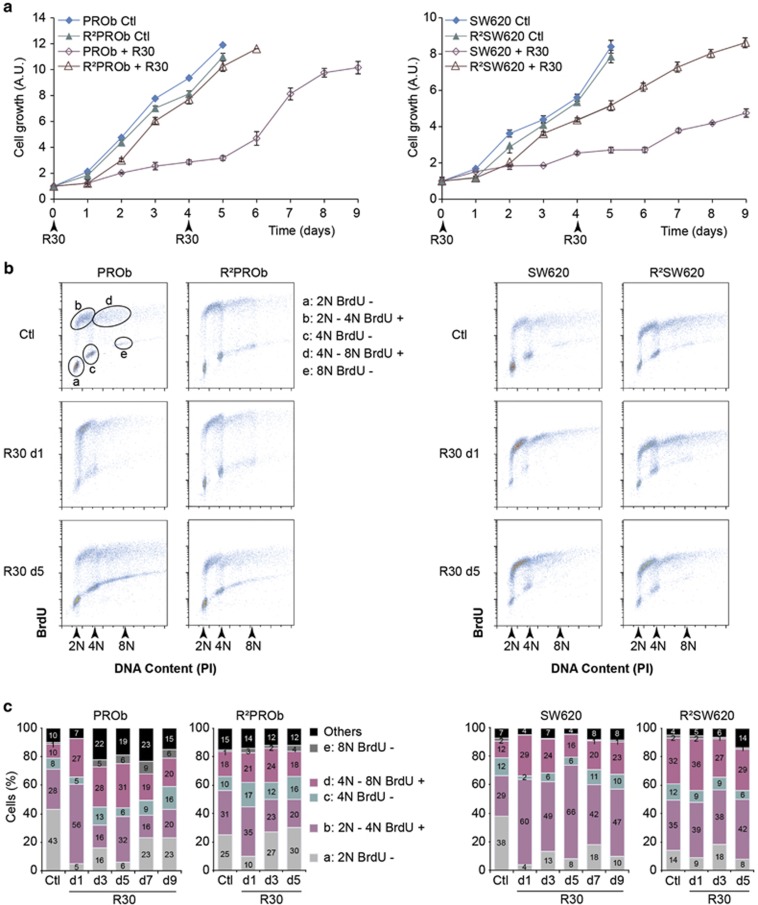
Repeated treatments with resveratrol lead to a polyploidisation and the emergence of a resistance. (**a**) Growth curves of colon cancer parental cells PROb and SW620 treated with 30 *μ*M of RSV (R30) at days 0 and 4 or left untreated (Ctl); proliferation of resistant cells R^2^PROb and R^2^SW620 obtained after 4-days pulses of R30 during two months is also presented. (**b**) Representative cell cycle analyses by PI/BrdU stainings of PROb and SW620 cells mock-treated (Ctl) or treated with R30 for 1 day (d1) and 5 days (d5). (**c**) Cumulative histograms (numbers shown are means) showing cell distribution in the different phases of their cell cycle after indicated times of R30 treatments in days (d). Flow cytometry gating procedure is depicted in B. Data are means±S.D. of three independent experiments

These observations led us to further characterise the long-term response of PROb and SW620 cells *in vitro*. We analysed the antiproliferative effects of RSV using a 30 *μ*M concentration which has been widely described to induce growth arrests and cell death of CCR cells^18, 24^ without causing cytotoxicity in normal cells^25^ (Supplementary Table 1). A single treatment of cells with RSV slowed down their proliferation for 4 days (Figure 4a). A second treatment at day four showed that RSV was not able to further inhibit cell growth, which restarted from day 6 for PROb and more progressively for SW620 cells, indicating that these cell lines became gradually resistant. To assess this phenomenon, we performed chronic treatments with RSV every 4 days for 2 months, resulting in the isolation of two stable populations, R^2^PROb and R^2^SW620 cells. According to their IC_50_ values, these populations were less sensitive towards RSV and they grew similarly to the parental cells even in the presence of R30 (Figure 4a, Supplementary Table 1).

To understand this escape, we analysed the effects of repeated RSV treatments on the cell cycle progression. RSV induced a rapid accumulation of parental cell lines in the S phases of the diploid and tetraploid cell cycles after 24 h (Figures 3b and c, denoted BrdU+). A second pulse at day 4 was less efficient in delaying S-phase progression, especially in PROb cells, showing a progressive loss of effects. Importantly, from three days of treatment, cells acquired a higher level of ploidy (Figures 4b and c, Supplementary Figure S1). Also, in the resistant R^2^PROb and R^2^SW620 populations, in which cell cycle progressions were not affected by RSV, the percentages of polyploid cells were higher than in parental cell lines. Loss of sensitivity towards RSV is therefore associated with an early and transient delay in S-phase progression and a robust generation of polyploid cells enriched in stable resistant populations.

### Resveratrol transiently induces cell death and senescence in colon cancer cells

**Figure 5:**
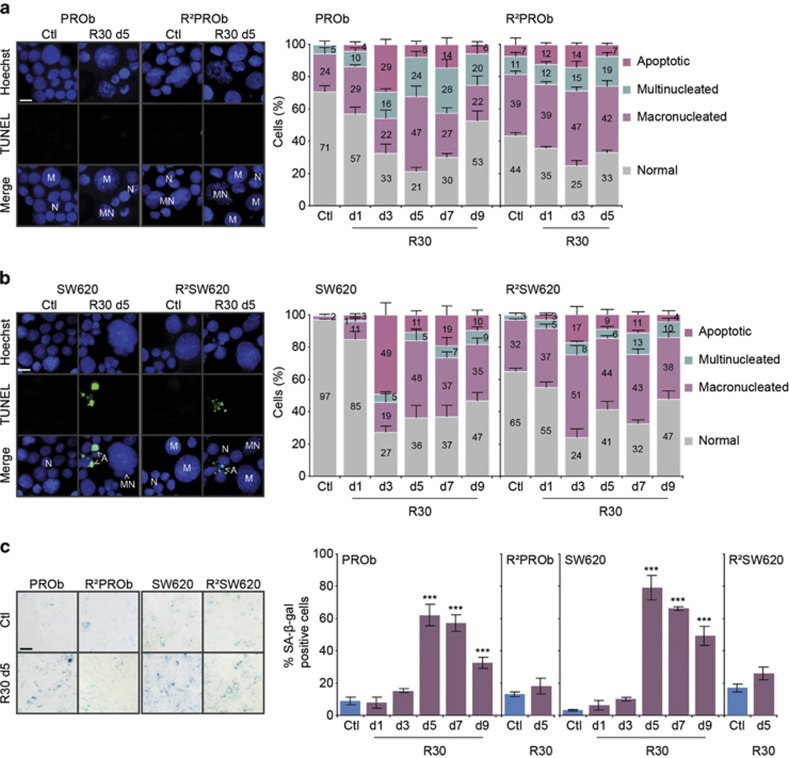
Resveratrol transiently induces cell death and senescence in colon cancer cells. (**a**,**b**) Analyses of the phenotypic responses of PROb and SW620 cells and of the isolated resistant towards RSV populations R^2^PROb and R^2^SW620. Cells were treated with 30 *μ*M of RSV (R30) for indicated times and analysed by Hoechst 33342 and TUNEL co-stainings. On the left, representative fields showing typical features observed by microscopic analyses of cytospun cells, cells with normal nuclei (N), macronucleated (M), multinucleated (MN) and apoptotic ones; *bar*, 20 *μ*m. On the right, cumulative histograms (numbers shown are means) showing the percentages of the different morphologies observed after indicated times of treatments in days (d) with R30 or mock-treated (Ctl). (**c**) Microscopic evaluation of SA-*β*-galactosidase activity in cells treated with R30. Representative microscopic observations are shown on the left after 5 days of R30 (d5); *bar*, 40 *μ*m. Percentages of SA-*β*-galactosidase positive cells after indicated times of R30 treatments in days are shown on the histograms on the right. Data in both panels are means±S.D. determined by counting 300 cells in three independent experiments. Statistical significance was determined by the Student's *t*-test with *P*<0.001 (***)

As chronic RSV treatment temporally induced apoptosis and senescence *in vivo* (Figure 1a), we precisely analysed the phenotypic consequences of long-term treatments *in vitro*. We found that RSV could induce three major nuclear phenotypes as compared with ‘normal' nuclei in terms of size and morphology: cells exhibiting enlarged nuclei denoted as macronucleated; multinucleated ones; and apoptotic cells with bright condensed or fragmented nuclei (Figures 5a and b). After three days, RSV-induced apoptosis in PROb (29%) and SW620 (49%) cells. Nevertheless, the second pulse of RSV at day four barely induced any apoptosis suggesting that apoptosis induction by RSV is only a transient effect. These results were confirmed by Annexin V / Propidium Iodide staining which showed the temporal induction of apoptosis and its progression (early apoptosis AV+/PI-; late apoptosis and secondary necrosis AV+/PI+), but no necrosis could be detected (AV-/PI+) (Supplementary Figure S2). Moreover, we found a transient increase of viable cells displaying macronucleations (peaking at 48% of cells at day 5), which could be linked to the progressive polyploidisation in duced by RSV (Figure 4c). In addition, a significant proportion of PROb cells became progressively multinucleated resulting in an amount of 28% of cells after 7 days of treatment, while this phenomenon was much reduced in SW620 cells. Macro- and multinucleated cells were mostly viable since they rarely appeared positive for TUNEL staining. Accordingly, we found high basal amounts of viable macro- and multinucleated cells in the isolated R^2^PROb- and R^2^SW620-resistant populations which correlated with their high level of polyploidy (Figure 4c). Finally, RSV-treated resistant cells displayed a very-low rate of apoptosis (Figures 5a and b, Supplementary Figure S2), and further increases of macro- and multinucleations were milder as compared with parental cells, showing that RSV did not really affect the phenotype of these populations.

Degrees of macrocytosis and multinucleation have been linked to drug-induced senescence, a phenotype we found in PROb tumours *in vivo* (Figure 1c). Our experiments demonstrated that RSV strongly but transiently induced senescence in CCR cells (Figure 5c). Senescence levels peaked at day 5, in accordance with the maximum level of macro- and multinucleated cells. As expected, these cells were almost all positive for the SA-*β*-galactosidase staining. Also, senescence induction was not lethal since no cell death was detected when this rate progressively decreased. Interestingly, RSV-treated resistant populations did not induce this mechanism, demonstrating that resistance implied a loss of sensitivity towards its pro-senescent activity.

Overall, we showed that repeated treatments with RSV of PROb and SW620 CCR cells firstly induced apoptosis then transient senescence in the remaining macro- and multinucleated viable cells.

### Resveratrol induces a short-term replication stress and subsequent DNA-damage signalling in colon cancer cell lines

**Figure 6:**
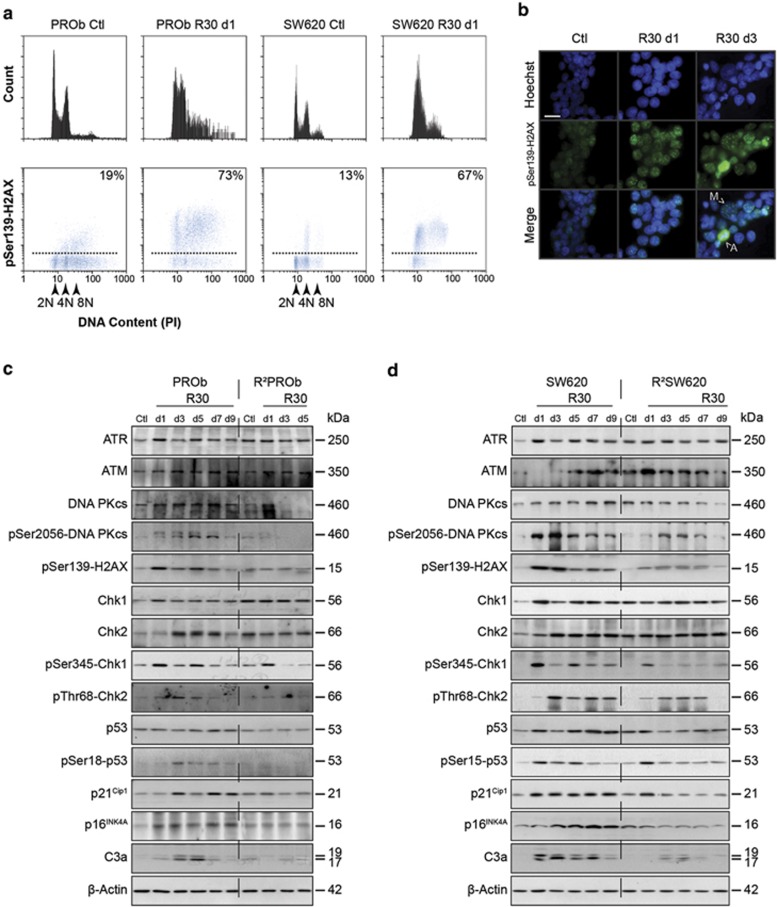
Resveratrol promotes the phosphorylation of the DNA-damage marker histone H2AX. (**a**) Flow cytometric analyses of *γ*H2AX/PI staining on PROb and SW620 cells after 1 day of treatment (d1) with 30 *μ*M of RSV (R30) or mock-treated (Ctl); the percentages represent the amounts of *γ*H2AX positive cells. (**b**) Microscopic analysis of *γ*H2AX immunostainings of PROb cells grown on coverslips treated with R30 during 1 (d1) or 3 days (d3). Arrows show macronucleated cells (M) and apoptotic ones (A); *bars*, on the left 100 *μ*m, on the right 20 *μ*m. (**c**, **d**) Time course of expression and phosphorylation of the components of the ATR, ATM and DNA-PK pathways were analysed by immunoblotting using the specified antibodies. PROb and SW620 cells as well as their resistant counterpart, R^2^PROb and R^2^SW620 populations, were treated with R30 for indicated times in days (d) and compared with the untreated cells (Ctl)

Resistance emergence towards RSV in CCR appeared to be related to a loss of sensitivity towards its pro-apoptotic and pro-senescence activities associated with a transient DDR induction *in vivo*. Thereafter, we took advantage of this new *in vitro* sensitive/resistant model to further characterise the DNA-damaging effects of RSV. To decipher the sequence of these events, we assessed the induction of *γ*H2AX in a cell cycle-dependent manner. After 24 h of treatment, ~70% of RSV-treated cells exhibited high levels of *γ*H2AX (Figure 6a). Most of the *γ*H2AX positive cells had diploid or tetraploid S-phase DNA contents, which is a hallmark of cells under replication stress. Microscopic analyses demonstrated typical *γ*H2AX foci after treatments and highly positive apoptotic cells as *γ*H2AX is known to be involved in this process (Figure 6b). Finally, as the *γ*H2AX induction appeared to be highly transient in RSV-treated resistant populations (Supplementary Figure S3), we hypothesised that RSV could generate a replication stress in sensitive cells which would ultimately adapt to this damaging effect.

To confirm this hypothesis, we further studied RSV effects on the regulation of the DDR in a time-dependent manner. In parental cell lines, a global and transient induction of these pathways was observed since DNA-PKcs, Chk1 and Chk2 were phosphorylated on activation sites (Figures 6c and d). In terms of time course, it was noticeable that Chk1 was activated prior to Chk2, consistent with a rapid replication stress/single strand break signalling which is mediated by ATR. Consequently, the main downstream mediator of these pathways, p53, was phosphorylated on its Ser15 (Ser18 in rodents), which was associated with an overexpression of p21^Cip1^ and p16^INK4A^. In resistant populations, these activations were compromised. Overall, these results showed that following an early replication stress, RSV induced a DDR in CCR cells concomitant with an S-phase delay and apoptosis followed by transient senescence in a long-term manner. Moreover, as the DDR activation was impaired in isolated resistant populations, we concluded that RSV anticancer properties highly depended on its DNA-damaging effects and subsequent cancer cell responses.

### DDR inhibitions enhance RSV anticancer activity in sensitive and resistant colon cancer cells

**Figure 7:**
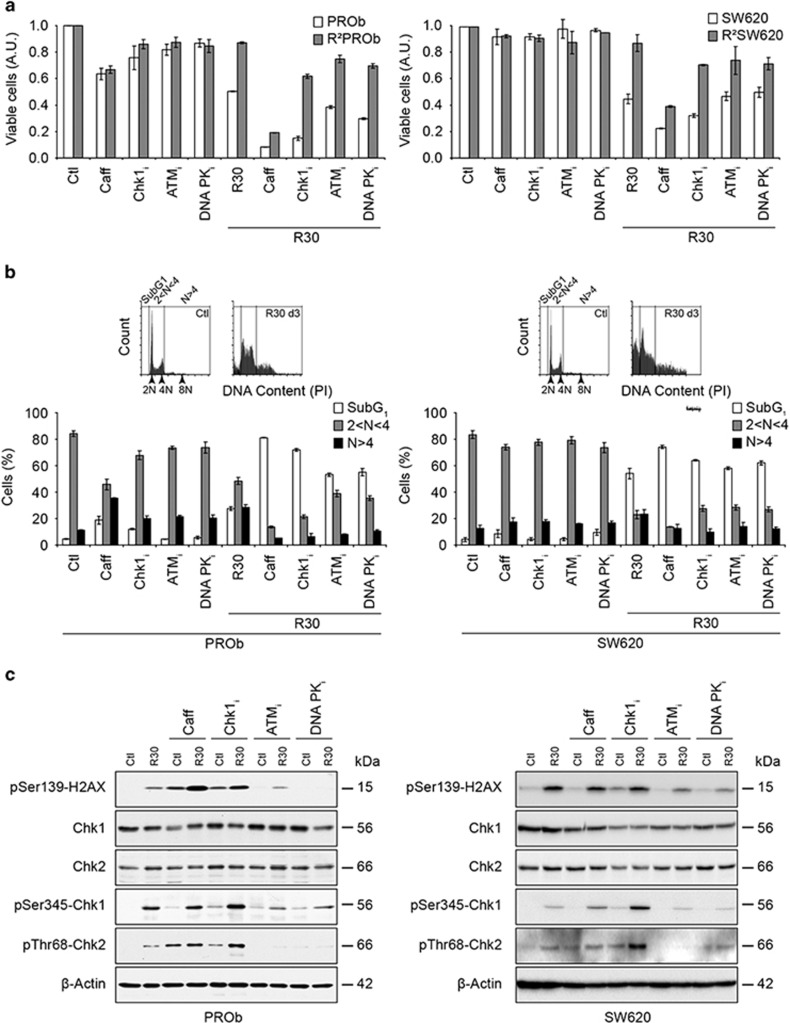
Resveratrol effects are linked to the activation of the DNA-damage response. (**a**) Sensitising effects of specific inhibitors of major kinases involved in the DNA-damage response on the growth of parental and resistant colon cancer cells treated with 30 *μ*m RSV (R30) for 24 h. Cells were pre-treated for 2 h with the PIKKs inhibitor caffeine (Caff), Chk1 inhibitor SB218078 (Chk1_i_), ATM inhibitor KU55933 (ATM_i_) or DNA-PK inhibitor NU7026 (DNA-PK_i_). (**b**) Cell cycle analysis by PI staining of cells treated for 72 h with R30 and with the different inhibitors as described before. Flow cytometry analysis was performed by gating SubG1, diploid (2N) and polyploid (>4N) cells as shown on the 1D plots. Data shown in A and B are means±S.D. of three independent experiments. (**c**) Levels of expression and phosphorylation of components of the DNA-damage response analysed by immunoblotting using the specified antibodies. PROb and SW620 cells were treated with the specified inhibitors as described above

To characterise the involvement of the DDR in RSV anticancer properties, we used specific kinases inhibitors. Caffeine, which inhibits all the PIKKs or SB218078 which targets Chk1, synergised with RSV in reducing CCR cells viability after 24 h, while ATM inhibitor KU55933 and DNA-PKcs inhibitor NU7026 did not (Figure 7a). After 72 h, pre-treatments with all these inhibitors more than doubled the level of apoptosis and highly reduced polyploidy (Figure 7b). These time-related effects of the inhibitors showed the implication of two axes of the DDR in response to RSV-induced replication stress, ATR-Chk1 then DNA-PK/ATM-Chk2 depicted above (Figure 6). Accordingly, after 72 h, caffeine or Chk1_i_ enhanced RSV-induced *γ*H2AX levels, which indicated cell cycle checkpoints abrogation, subsequent lack of DNA-damage repair (shown by enhanced Chk2 phosphorylation, Figure 7c) and apoptosis (Figure 7b). Then, inhibitions of these kinases partly restored RSV activity towards resistant cells showing that their lack of response might be linked to their ability to react to RSV-induced DNA damage (Figure 7a). Interestingly, resistant cells were not more sensitive to other DNA-damaging agents used to treat CCR, since we did not detect any cross-resistance towards 5-fluoro-uracil, oxaliplatin and SN-38 (Supplementary Table S1). However, the antiproliferative effects of doxorubicin were compromised in cells resistant towards RSV showing that these two compounds might share common mechanisms of action. Altogether, these results demonstrated that RSV-induced DDR and subsequent checkpoint activation in sensitive cells was responsible for its transient anticancer effects. They also show that prolonged RSV treatments and subsequent resistance emergence directly or indirectly depends on its DNA-damaging effect.

### Reactive oxygen species mediate RSV-induced DNA damage and subsequent effects

**Figure 8:**
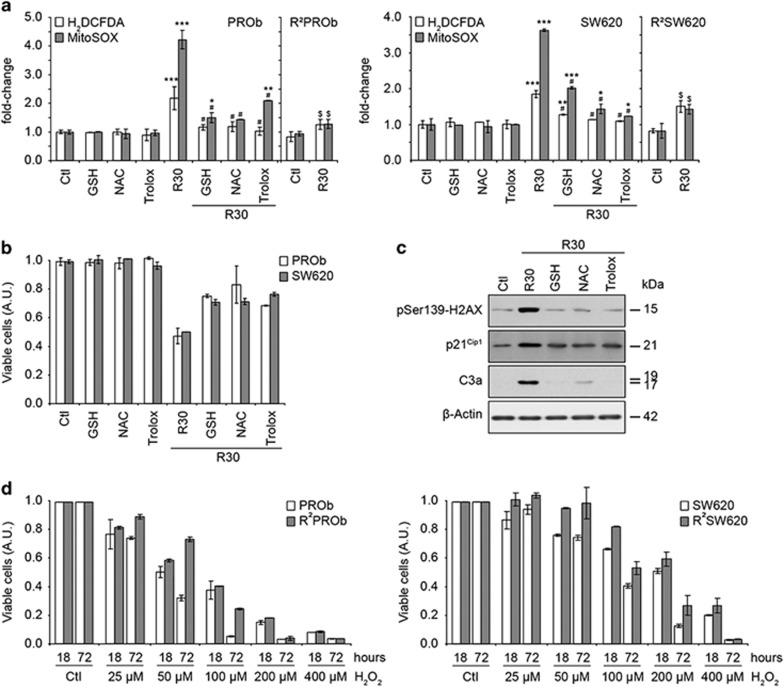
Reactive oxygen species mediate resveratrol-induced DNA damage and subsequent effects. (**a**) Levels of reactive oxygen species (ROS) in PROb and SW620 cells as well as on the resistant populations R^2^PROb and R^2^SW620 analysed by flow cytometry after 24 h of treatment with 30 *μ*M of resveratrol (R30). The probes used were H_2_DCFDA and MitoSOX which respectively detect the global level of ROS and the O_2_^−^ produced by mitochondria. Cells were also grown in the presence of the antioxidant-reduced glutathione (GSH), *N*-Acetylcystein (NAC) and Trolox. (**b**) Effects of the supplementation of media with the antioxidants described above on the viability of cells treated with R30 for 24 h. (**c**) Effects of co-treatments of PROb cells with R30 and the antioxidants described above on the activation of H2AX and caspase-3 and on the expression of p21^Cip1^ assayed by immunoblotting. (**d**) Antiproliferative activity of H_2_O_2_ on colon cancer cells was determined by treatments with the indicated concentrations of H_2_O_2_ for 18 h and after release in fresh media for 72 h. Data shown in A, B and D are means±S.D. of three independent experiments

ROS are known to induce replication stress and DNA damage^26^ and are implicated in the anticancer effects of potent chemotherapeutic drugs (e.g. doxorubicin). RSV is usually described as an antioxidant, but as it induced replication stress and shares properties with doxorubicin, it seemed important to study the role of ROS in RSV effects. Thus, using a H_2_DCFDA probe, we found that RSV efficiently induced an overproduction of ROS in CCR cells (Figure 8a). Furthermore, mitochondrial O_2_^-^ was measured using a MitoSOX probe and was found to be overproduced. These productions were counteracted by antioxidants like reduced glutathione, *N*-Acetylcystein and Trolox. Moreover, these antioxidants partly inhibited RSV antiproliferative effects (Figure 8b). Antioxidants also counteracted the DNA-damaging effects of RSV as well as apoptosis induction as revealed by *γ*H2AX, p21 and cleaved caspase-3 immunoblots (Figure 8c). Interestingly, RSV-induced ROS production was highly impaired in resistant populations showing an important adaptation towards the oxidative stress after prolonged treatments (Figure 8a). To assess this adaptation, we analysed the antiproliferative effects of H_2_O_2_ and showed that resistant cells were obviously less sensitive to oxidative stress (Figure 8d). Accordingly, we detected a loss of sensitivity of these cells towards doxorubicin, which is known to partly damage cells through an oxidative stress (Supplementary Table S1). Altogether, these results demonstrated the importance of RSV-induced ROS production on its indirect DNA-damaging effects, subsequent transient anticancer effects and a long-term adaptation of cancer cells towards oxidative stress.

## Discussion

In the present report, we studied the effects of RSV towards CCR models *in vivo* and *in vitro*. We found that RSV shares common properties with DNA-damaging drugs widely used to treat cancer by inducing similar mechanisms of resistance establishment. ^11, 27^ We subsequently showed that this was related to an overproduction of ROS against which CCR progressively developed adaptive mechanisms.

It was initially reported that RSV reduces the tumour progression in *in vivo* cancer models^28, 29^ but these results were challenged by several groups showing no beneficial effect of RSV. Many factors may be involved in these controversial results, including the sensitivity of the models, the doses and administration routes and the animal species used which could show various immune properties.^30, 31, 32, 33^ One of the main arguments is still the low bioavailability of RSV. More recently, studies by Patel *et al.*^21^ and Aires *et al.*^22^ showing that RSV metabolites accumulate in colon and exert anticancer activity seem to corroborate the positive argument. In the present report, pharmacological administrations of RSV were not able to counteract the growth of CCR *in vivo*, although plasma concentrations of free RSV and its metabolites were comparable with previous studies.^34, 35^ In fact, we showed that RSV could induce DNA damage in CCR as recently reported in head and neck squamous carcinoma.^36^ For the first time, we showed that RSV triggered a temporal related DDR *in vivo* as well as its phenotypic consequences, that is, senescence and apoptosis. In *in vitro* studies, CCR cells transiently accumulated in S-phase of the diploid and tetraploid cell cycle and it is now obvious that tetraploid populations must be considered as they are often more resistant towards chemotherapies.^37^ A recent report using tetraploid cells isolated after mitotic inhibitors exposure has shown that RSV efficiently killed these cells, but these models do not mimic RSV-induced polyploidisation by itself.^38^ Indeed, the present report demonstrates that long-term treatments of CCR cells with RSV led to the emergence of polyploid populations which showed resistance towards its anticancer effects. We have already described the occurrence of similar phenomena with the DNA-damaging agent cisplatin, which led to resistance emergence through endoreduplication along with an induction of senescence and a reversible polyploidisation.^39^

Previous reports have described a pro-senescence activity of RSV towards cell lines^11, 40^ which were blocked in senescence in a p53-dependent manner. In this study, cells escaped this state despite the fact that they both display a p53 transactivational activity (PROb cells are p53^wt^ and SW620 cells are overexpressing a p53^mut^ active form). p53 may be one of the main factor implicated in RSV cell line-dependent potential as its level and time of activation have been recently shown to regulate the senescence/quiescence/apoptosis balance^41^. Thus, this study demonstrates that RSV induces more apoptosis in SW620 cells carrying a hyperactive p53^mut^. Moreover, the transient senescence response observed is comparable with studies reporting the implication of senescence in the resistance mechanisms triggered by cancer cells towards DNA-damaging drugs.^39, 42, 43^

We next demonstrated that RSV anticancer effects were linked to its ability to induce the DDR as shown by the rapid activation of *γ*H2AX. This was concomitant with a rapid induction of a Chk1-related response and a delayed Chk2 activation, leading to an overall p53-dependent response. Inhibitors of DDR-implicated kinases demonstrated that subsequent checkpoint and repair activations were responsible for the maintenance of the viable polyploid cells, since their inhibitions dramatically reduced polyploidy and sensitised cells to apoptosis. Moreover, the early Chk1 response was consistent with the occurrence of a rapid replication stress induction and its inhibition synergised with RSV in inducing DNA damage and apoptosis as previously shown for other chemotherapies.^44^ Importantly, the present report shows that RSV's indirect DNA damaging properties and subsequent anticancer properties are mostly related to an induction of ROS production as shown by co-treatments with antioxidants. This statement was reinforced by the rapid induction of ATM, p16 and p21 expressions, which have been recently reported to participate in the crosstalk between ROS production and senescence.^45, 46, 47^

The present work raises some doubts about the widely accepted antioxidant potential of RSV. There is now a compelling evidence that this overall antioxidant effect is probably an indirect consequence of its primary pro-oxidant potential. Similar observations have been published with other polyphenols like epigallocatechin-3-gallate and curcumin, linking their DNA damage-like effects to their potential to induce ROS production.^48, 49^ Here, populations of cells which have been exposed to RSV during several weeks became more resistant to H_2_O_2_ and doxorubicin (known to trigger oxidative stress), and it is tempting to hypothesise that detoxifying systems are involved as shown for other potent chemopreventive molecules.^50^

To conclude, this study provides new insights into RSV as an anticancer compound linking its *in vitro* effects to its potency *in vivo*, and collectively these experiments suggest that a potential acquired resistance towards RSV during prolonged treatment should be taken into account in the case of clinical trials.

## Materials and Methods

### Chemical reagents and antibodies

*Trans*-RSV (>99% purity) was obtained from Tianjin Jianfeng Natural Products (Tianjin, China). All other chemicals were obtained from Sigma Aldrich (Saint Quentin-Fallavier, France) unless stated otherwise. Antibodies used are described in the Supplementary Table S2.

### Cell lines

The PROb cell line (DHD-K12-TRb, deposited at the European Collection of Animals Cell Culture, ECACC, Salisbury, UK), obtained from a well-differentiated colon carcinoma induced in the BD-IX rat strain^51^, was cultured in RPMI 1640 medium supplemented with 10% foetal calf serum. The human colon carcinoma SW620 and the normal rat intestinal IEC18 cell lines (ECACC) were maintained in Dulbecco's modified Eagle's medium containing 10% foetal calf serum.

### Cell treatments

Cells were treated for indicated times with indicated concentrations of RSV dissolved in the media containing a final amount of 0.1% ethanol or mock-treated. RSV-resistant PROb and SW620 cells, named R^2^PROb and R^2^SW620, respectively, were obtained by culturing exponentially growing parental cell lines in a selective pressure of 30 *μ*M RSV during 2 months, resulting in RSV-resistant populations stable in time. To determine the involvement of the DNA-damage pathways in RSV-mediated effects, cells were either pre-treated for 2 h prior to RSV treatment with 2 mM caffeine known to inhibit the PIKKs, 2 *μ*M of the Chk1 inhibitor SB218078, 10 *μ*M of the ATM inhibitor KU55933 or 10 *μ*M of the DNA-PK inhibitor NU7026, all purchased from Merck Chemicals (Nottingham, UK). To counteract RSV-induced ROS, cells were pre-treated for 4 h with 10 mM of reduced glutathione or *N*-Acetylcystein (a glutathione precursor), or 10 *μ*M of Trolox, a water-soluble derivative of vitamin E. H_2_O_2_ antiproliferative activity was determined by treating cells with concentrations from 25–400 *μ*M for 18 h and after a release in fresh medium for 72 h.

### Proliferation assays

Cells were seeded in quadruplicate in 24-well plates and treated with indicated concentrations of chemicals 24 h later. After the indicated times, cells were washed with PBS, stained with crystal violet (0.5% (w/v) crystal violet in 25% (v/v) methanol) for 5 min and gently rinsed with water three times. Absorbances were read at 540 nm after extraction of the dye by 0.1 M sodium citrate in 50% ethanol.

### Flow cytometry

Cell cycle analyses by BrdU incorporation were conducted as previously described.^18^ For H2AX phosphorylation on Ser139 analyses, cells were fixed in 100% methanol on ice for 15 min, washed in PBS, blocked for 20 min in PBS-1% BSA and incubated with a rabbit anti-pSer139-H2AX antibody (1/1000 in PBS-BSA, Santa-Cruz Biotechnology, CA, USA) for 1 h. After washes in PBS, cells were incubated for 1 h with a secondary anti-rabbit Alexa-488 antibody (1/500 in PBS-BSA, Invitrogen/Molecular Probes, Eugene, OR, USA). Cells were then washed and the DNA counterstained with 50 *μ*g/ml propidium iodide for 30 min in the presence of 200*-μ*g/ml RNAse A. To measure the production of ROS, we used the probes 2′,7′-dichlorodihydrofluorescein diacetate (H_2_DCFDA) which fluoresces when oxidised by intracellular ROS and MitoSOX which responds to mitochondrial O_2_^-^ (both obtained from Molecular Probes). Briefly, cells were incubated with 10*-μ*m H_2_DCFDA or MitoSOX during 15 min, washed with PBS and immediately analysed by flow cytometry. All the assays were monitored on a Galaxy flow cytometer (Partec, Münster, Germany) and analysed with the FloMax software (Partec) on at least 100 00 cells.

### Fluorescence microscopy

TdT-mediated dUTP Nick-End Labeling (TUNEL) and Hoechst 33342 stainings were used to analyse nuclear morphologies according to the manufacturer's instructions (DeadEnd Fluorometric TUNEL System, Promega, Charbonnières-les-Bains, France). For *in vitro* assays, adherent and floating cells were cytospun and phenotypes of nuclei were determined by analysing at least 300 cells per experiment. For *in vivo* staining, tumours were cut in 6*-μ*m sections using a cryostat microtome, mounted on gelatinised slides, processed and analysed by determining the mean pixels intensities of random microscopic fields by using the ImageJ software (NIH).

### Senescence-associated *β*-galactosidase (SA-*β*-gal) determination

Stainings for SA-*β*-gal activity revealed at pH 6.0 were performed as previously reported.^52^ *In vitro*, cells were grown in 12-well plates on glass coverslips and treated with RSV for the times indicated. *In vivo*, slides were analysed as described above.

### Tumour growth analyses *in vivo*

Exponentially growing PROb and SW620 cells were harvested and 10^6^ (PROb) or 3 × 10^6^ (SW620) cells were injected subcutaneously into syngeneic BD-IX rats (PROb) or into NMRI-nu Nude mice (SW620; Janvier Labs, Saint-Berthevin, France). Animals were individually treated *per os* by administrating an appropriate amount of RSV. Amounts of RSV were weekly adapted to corporal mass to obtain 40, 100 or 200 mg/kg per day treatments. Treatments began at different tumour stages, from day 2 after cells inoculation and from day 14. Tumour volumes were evaluated weekly using a calliper. Animal use and handling were approved by the local veterinary committee and performed according to European laws for animal experimentation.

### Resveratrol plasma concentration measuremnts

Plasma samples were treated for 2 h at 37 °C with 200 U of *ß*-glucuronidase (HP-2 type) in 0.5 ml of 0.2 M acetate buffer; control samples were incubated with buffer alone. Each sample was then acidified to pH 4.5, received carbamazepine as internal standard, and was passed through a C18 Sep-Pak cartridge (Waters, Milford, MA, USA) pre-conditioned with methanol and acetate buffer. After washing with acetate buffer, RSV and/or its metabolites were eluted with 2 ml of methanol. After evaporation, they were redissolved in ethanol for analysis. RSV assays were performed on a reverse-phase Nucleosil C18 column (250 × 4.6 mm, 5 *μ*m) from Alltech Associates (Deerfield, IL, USA) in a Waters 625-LC system. Compounds were eluted from the column with a gradient containing water and acetonitrile. The UV detector (Waters 486, Milford, MA, USA) was set at 306 nm. RSV amounts were quantified with an SP4400 ChromJet integrator (Spectra Physics, San Jose, CA, USA).

### Western blotting

After the indicated treatments, adherent and floating cells were washed in PBS, lysed in a Ripa buffer and western blot analyses were performed as previously described.^18^

### Statistical analysis

The *in vitro* data are expressed as the mean±S.D. of three independent experiments. *In vivo* data are shown as median±the first and the third quartiles. Statistical significances were evaluated either with the Mann–Whitney or the *t*-test as required.

## Abbreviations

ATM: Ataxia Telangiectasia Mutated
ATR: ATM and Rad3-related
CCR: Colorectal Cancer
Chk1: Checkpoint kinase 1
Chk2: Checkpoint kinase 2
DDR: DNA Damage Response
DNA-PKcs: DNA-dependent Protein Kinase, catalytic subunit
PIKKs: Phosphatidylinositol 3-kinase-related kinases
ROS: Reactive Oxygen Species
RSV: Resveratrol
SA-*β*-galactosidase: Senescence-Associated *β*-galactosidase
TRAIL: Tumour-necrosis-factor Related Apoptosis Inducing ligand
TUNEL: TdT-mediated dUTP Nick-End Labelling.
