# Supplementary material for: The role of reactive oxygen species and subsequent DNA-damage response in the emergence of resistance towards resveratrol in colon cancer models
Source: Cell Death Dis. 2014 Nov 20;5(11):e1533–. doi: 10.1038/cddis.2014.486 (PMC4260744; doi:10.1038/cddis.2014.486)
Supplement: Supplementary Information [file cddis2014486x1.pdf]

SUPPLEMENTAL INFORMATION

Supplemental Table 1. IC50 (72 h) of trans-resveratrol (RSV), 5-Fluoro-uracil (5-FU), Oxaliplatin (OxPt), SN38 and Doxorubicin (Dox) against parental PROb and SW620 cell lines, RSV-resistant populations R²PROb and R²SW620, and against the rat normal intestinal IEC18 cell line.

IC_{50}	RSV (µM)	5-FU (µM)	OxPt (nM)	SN38 (nM)	Dox (nM)
PROb	26.2 ± 0.8	1.62 ± 0.21	103.3 ± 5.3	15.5 ± 5.3	115.5 ± 16.1
R²PROb	69.0 ± 2.4 (**)	2.03 ± 0.28	101.4 ± 10.1	25.8 ± 4.8	212.1 ± 23.5 (*)
SW620	23.6 ± 0.7	2.07 ± 0.46	302.2 ± 73.1	2.0 ± 0.4	3.1 ± 0.8
R²SW620	50.6 ± 4.3 (**)	3.29 ± 0.62	445.2 ± 104.2	3.2 ± 0.3	11.5 ± 3.2 (**)
IEC18	82.3 ± 5.5	4.12 ± 0.54	391.8 ± 55.3	7.2 ± 0.8	22.0 ± 1.4

Supplemental Table 2. Antibodies used for western blot (WB) and immunofluorescence analyses (IF).

Antigen	Manufacturer	Reference	Dilution
β-Actin	Sigma-Aldrich	A3853	1/10000 (WB)
ATM	Santa-Cruz Biotechnology	sc-7129	1/200 (WB)
ATR	Santa-Cruz Biotechnology	sc-1887	1/200 (WB)
Chk1	Santa-Cruz Biotechnology	sc-8408	1/500 (WB)
pSer345-Chk1	Cell Signaling	#2341	1/1000 (WB)
Chk2	Upstate (Millipore)	05-649	1/1000 (WB)
pThr68-Chk2	Abcam	ab3501	1/1000 (WB)
Cleaved Caspase-3	Cell Signaling	#9661	1/1000 (WB)
DNA PKcs	Abcam	ab230	1/2000 (WB)
pSer2056-DNA PKcs	Abcam	ab18192	1/1000 (WB)
pSer139-H2AX	Santa-Cruz Biotechnology	sc-101696	1/500 (WB); 1/1000 (IF)
$p16^{INK4A}$	Santa-Cruz Biotechnology	sc-1661	1/200 (WB)
$p21^{Cip1}$	Santa-Cruz Biotechnology	sc-397	1/500 (WB)
p53	Cell Signaling	#2524	1/1000 (WB)
pSer15-p53	Cell Signaling	#9284	1/1000 (WB)

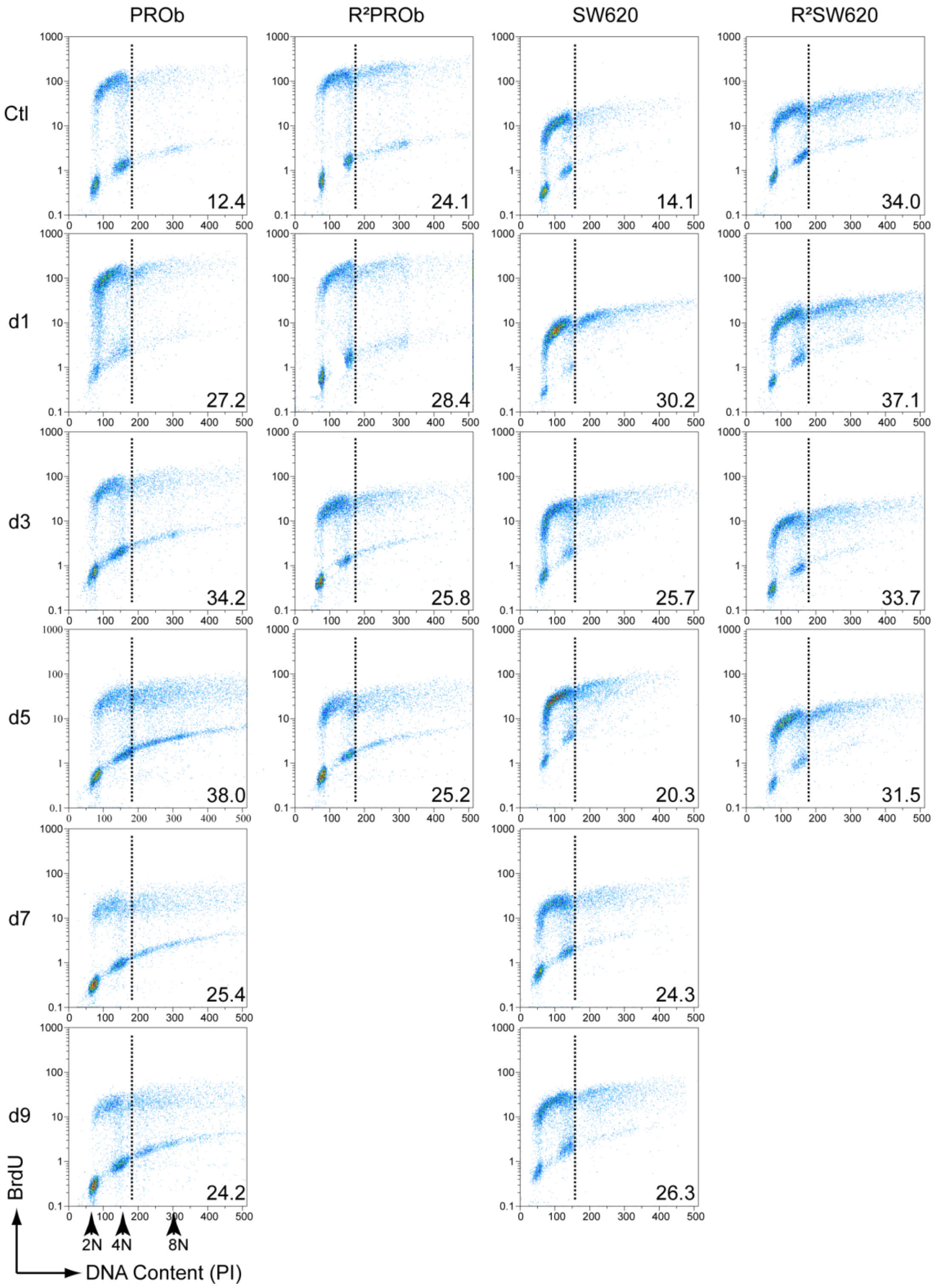

Supplemental Figure 1. Repeated treatments with RSV lead to polyploidisation.

Representative cell cycle analyses by PI/BrdU stainings of PROb, SW620 and resistant towards RSV populations mock-treated (Ctl) or treated with R30 for n days (d). Values are percentages of polyploid cells (>4N).

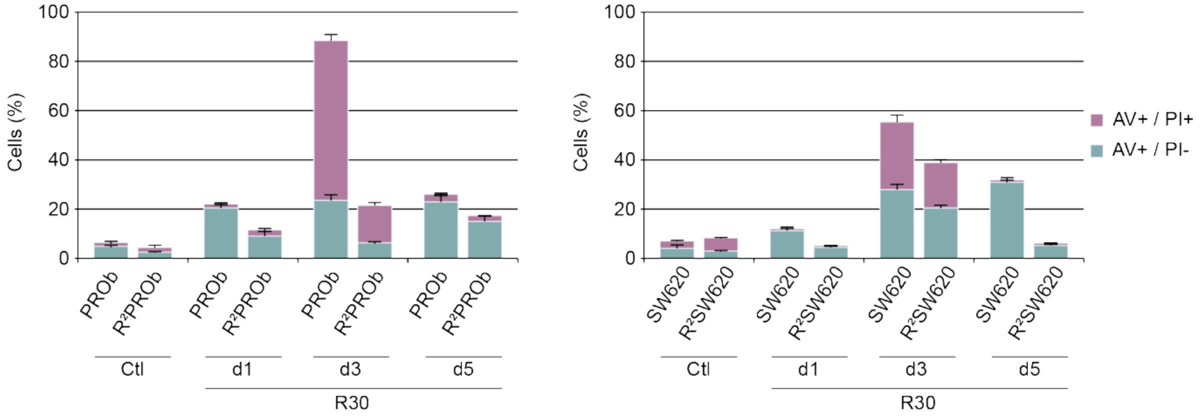

Supplemental Figure 2. Resveratrol transiently induces cell death in colon cancer cells.

Percentages of dead cells analysed by Annexin V / Propidium Iodide staining and flow cytometry. PROb, SW620 and resistant towards resveratrol R²PROb and R²SW620 colon cancer cells were treated with 30 µM or resveratrol (R30) or mock-treated (Ctl) for one to five days (d1, d3 and d5), then processed and analysed according to the manufacturer (BD Biosciences). Cumulative histograms show the percentages of cells positive for Annexin V (AV+) and their Propidium Iodide (PI) status. Values are means ± SD of three independent experiments.

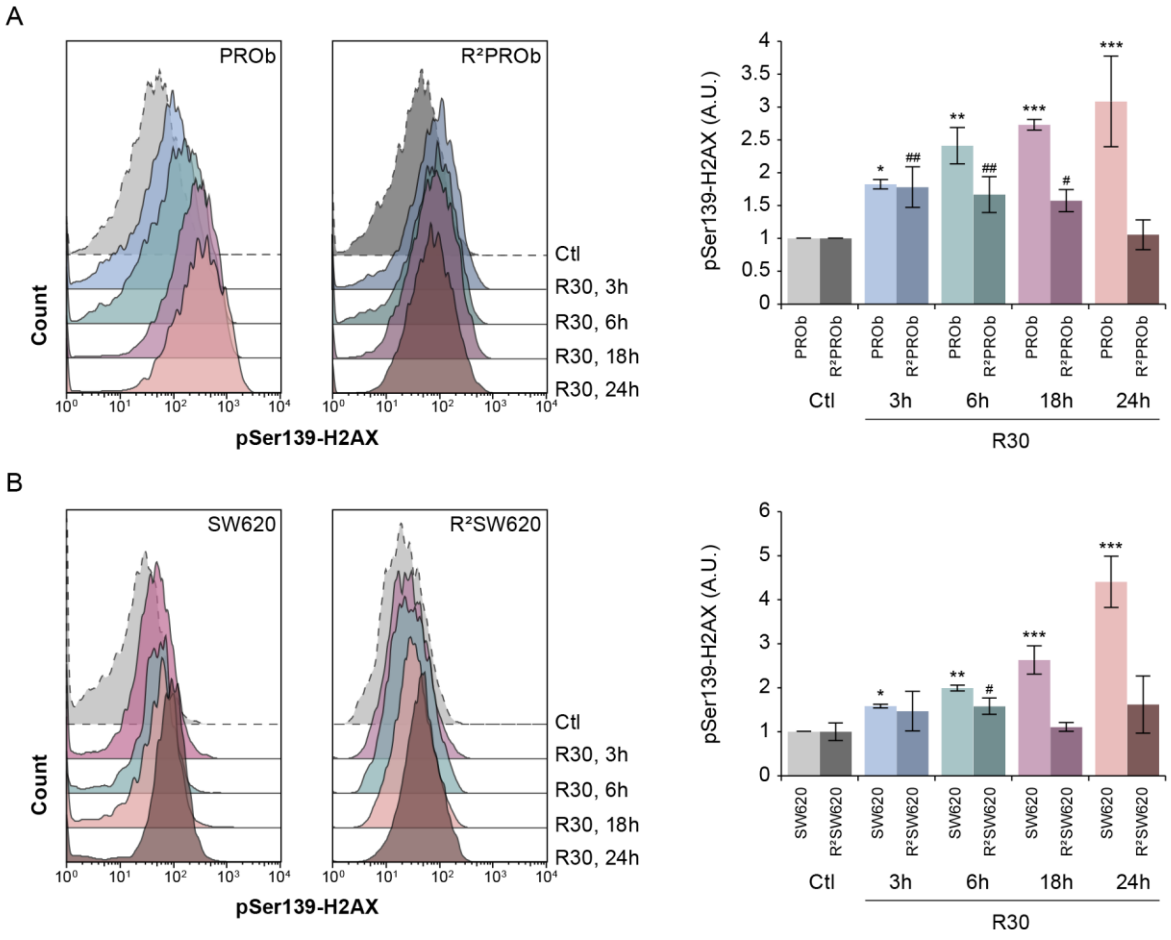

Supplemental Figure 3. RSV-induced short-time activation of pSer139-H2AX in sensitive parental PROb and SW620 and in resistant R²PROb and R²SW620 populations.

Flow cytometric analyses of γH2AX/PI staining on PROb (A) and SW620 (B) cells after 3 to 24 hours treatment (h) with 30 µM of resveratrol (R30) or mock-treated (Ctl). Representative 1D-plots are shown on the left. On the right, data are mean fluorescence intensities related to controls ± SD of three independent experiments. Statistical significance was determined by the Student's t-test with $P<0.05$ (*), $P<0.01$ (**) and $P<0.001$ (***) determined *vs* parental PROb or SW620 cells, or *vs* Ctl resistant R² cells with $p<0.05$ (#), $p<0.01$ (##).
